# Type I Interferon Activates PD-1 Expression through Activation of the STAT1-IRF2 Pathway in Myeloid Cells

**DOI:** 10.3390/cells13131163

**Published:** 2024-07-08

**Authors:** Liyan Liang, Yingcui Yang, Kaidi Deng, Yanmin Wu, Yan Li, Liya Bai, Yinsong Wang, Chunwan Lu

**Affiliations:** 1 School of Life Sciences, Tianjin University, Tianjin 300072, China; lly_lly@tju.edu.cn (L.L.); 2021226047@tju.edu.cn (Y.Y.); dkd1226@tju.edu.cn (K.D.); wym971220@163.com (Y.W.); yanli_@tju.edu.cn (Y.L.); 2 School of Pharmacy, Tianjin Medical University, Tianjin 300070, China; bailiya@tmu.edu.cn (L.B.); wangyinsong@tmu.edu.cn (Y.W.)

**Keywords:** PD-1, myeloid cells, IFNβ, pSTAT1, IRF2

## Abstract

PD-1 (Programmed cell death protein 1) regulates the metabolic reprogramming of myeloid-derived suppressor cells and myeloid cell differentiation, as well as the type I interferon (IFN-I) signaling pathway in myeloid cells in the tumor microenvironment. PD-1, therefore, is a key inhibitory receptor in myeloid cells. However, the regulation of PD-1 expression in myeloid cells is unknown. We report that the expression level of PDCD1, the gene that encodes the PD-1 protein, is positively correlated with the levels of IFNB1 and IFNAR1 in myeloid cells in human colorectal cancer. Treatment of mouse myeloid cell lines with recombinant IFNβ protein elevated PD-1 expression in myeloid cells in vitro. Knocking out IFNAR1, the gene that encodes the IFN-I-specific receptor, diminished the inductive effect of IFNβ on PD-1 expression in myeloid cells in vitro. Treatment of tumor-bearing mice with a lipid nanoparticle-encapsulated IFNβ-encoding plasmid (IFNBCOL01) increased IFNβ expression, resulting in elevated PD-1 expression in tumor-infiltrating myeloid cells. At the molecular level, we determined that IFNβ activates STAT1 (signal transducer and activator of transcription 1) and IRFs (interferon regulatory factors) in myeloid cells. Analysis of the cd279 promoter identified IRF2-binding consensus sequence elements. ChIP (chromatin immunoprecipitation) analysis determined that the pSTAT1 directly binds to the irf2 promoter and that IRF2 directly binds to the cd279 promoter in myeloid cells in vitro and in vivo. In colon cancer patients, the expression levels of STAT1, IRF2 and PDCD1 are positively correlated in tumor-infiltrating myeloid cells. Our findings determine that IFNβ activates PD-1 expression at least in part by an autocrine mechanism via the stimulation of the pSTAT1-IRF2 axis in myeloid cells.

## 1. Introduction

Programmed cell death protein 1 (PD-1, also known as CD279 and PDCD-1) is an inhibitory receptor on T cells. PD-1 is undetectable in naïve T cells, but rapidly activated upon T cell receptor (TCR)-mediated T cell activation. Engagement of PD-1 on T cells by its physiological ligands, programmed cell death protein 1 ligand 1 (PD-L1, also known as CD274, or B7-H1) or programmed cell death protein 1 ligand 2 (PD-L2, also known as CD273, or B7-DC), induces T cells into a dysfunctional state known as exhaustion, and thus serves as an immune checkpoint to prevent autoimmunity under physiological conditions [1,2,3,4,5]. Under pathological conditions such as cancer, PD-L1 is highly elevated on tumor cells and immune cells in human cancer patients and tumor-bearing mice, and binds to PD-1 to repress T cell activation, resulting in tumor cell immune escape from host immune surveillance [6,7,8,9,10,11]. Consequently, immune checkpoint inhibitors have been developed to block PD-1/PD-L1 interactions to reverse tumor-induced immune suppression and inhibit tumor progression [12,13]. PD-1 is therefore a promising target in human cancer immunotherapy. The regulation of PD-1 expression in T cells has been an active research area for the last decade [12,13,14,15,16,17,18,19,20] and has been extensively studied in different cellular contexts [21,22,23,24,25].

In T cells, PD-1 transcription is regulated by dynamic interplays between epigenetic modifiers and transcription factors at its promoter region, which varies according to the T cell state and the cellular microenvironment [17,18,19,26,27]. Demethylation and histone modifications of the *Pdcd1* promoter regulatory region following TCR engagement mediate transcription factor association with the *Pdcd1* promoter DNA and subsequent *Pdcd1* transcription [19,26,28]. Multiple transcription factors, including BLIMP-1, Eomes, FOXO1, NFATc1, NF-κB, STAT3, STAT4 and T-Bet have been shown to directly regulate PD-1 expression in T cells at various cellular contexts in virus-infected and tumor-bearing hosts [15,17,27,28,29,30,31,32]. These transcription regulation mechanisms define PD-1 expression’s upregulation and T cell function under physiological and pathological conditions, and provide the basis for the development of immune checkpoint inhibitor immunotherapy for human diseases such as cancer [1,11,33].

Although PD-1 is primarily expressed in activated and exhausted T cells, emerging experimental data indicate that PD-1 is also expressed in myeloid cells and plays a critical role in myeloid cell activation and differentiation [34,35,36]. Deleting PD-1 only in myeloid cells leads to metabolic reprogramming and the differentiation of myeloid-derived suppressor cells (MDSCs), resulting in increased effector memory T cells and decreased tumor growth despite preserved PD-1 expression in T cells in tumor-bearing mice [36]. Furthermore, PD-1 regulates myeloid differentiation and monocytic dendritic cell (moDC) lineage commitment by repressing the expression of HOXA10 and IRF8 to restrain myelocyte differentiation to suppress anti-tumor immunity [34]. In addition, tumor cell PD-L1 can bind myeloid cell PD-1 to activate SHP-2. The activated SHP-2 then represses IFN-I expression in myeloid cells to impair the IFN-I signaling pathway. Decreased IFN-I signaling pathway results in decreased Cxcl9/10 expression, leading to impaired T cell tumor recruitment to promote tumor immune evasion [35]. It is therefore likely that myeloid cell PD-1 might be a key mechanism of anti-tumor immunity mediated by ICI (immune checkpoint inhibitor) immunotherapy [36]. PD-1 expression in myeloid cells therefore is a critical immune suppression mechanism underlying tumor immune evasion. Nevertheless, the mechanism underlying PD-1 expression regulation in myeloid cells is incompletely understood. 

We report here that the *PDCD1* transcription level exhibits a positive correlation with the *IFNB1* and *IFNAR1* level in myeloid cells in human colorectal cancer patients. Furthermore, IFNβ upregulates PD-1 expression in mouse myeloid cells both in vitro and in vivo. Mechanistically, we determine that IFNβ induces pSTAT1 binding to the *irf2* promoter and IRF2 binding to the *cd279* promoter to activate PD-1 transcription.

## 2. Materials and Methods

**Patient Dataset Analysis.** *PDCD1*, *IFNB1*, *IFNAR1*, *STAT1* and *IRF2* single cell RNA-seq datasets of human colorectal cancer and all correlations were extracted from the GEO database (GSE178341) [37].

**Mice.** BALB/c mice (female, 7–8 weeks old) were purchased from Huafukang, Beijing, China. All studies involving the use of mice were covered by a protocol approved by the Institutional Animal Care and Use Committee of Tianjin University (Approval #TJUE-2021-016. Approval date: 1 March 2021). 

**Cell Lines.** Mouse monocyte-macrophage leukemia cell line RAW264.7 and murine colon tumor cell line CT26 were obtained from Meilunbio, Dalian, China. Bone marrow cells were isolated from BALB/c mice and treated with 2 ng/mL recombinant mouse GM-CSF (Cat# P00195, Solarbio, Beijing, China) to be induced as bone marrow-derived MDSCs. All cell lines were tested every month for mycoplasma, and all cell lines used in this study were mycoplasma-free at the time of the study.

**Cationic Lipid Nanoparticle and Plasmid**. DOTAP(N-[1-(2,3-dioleoyloxy)-propyl]-N, N, N-trimethyl ammonium methyl-sulfate)-cholesterol (1:1 molar ratio) was synthesized as previously stated [38]. To prepare lipid nanoparticle-encapsulated DNA, plasmid DNA and DOTAP-Cholesterol were diluted in 5% glucose (kindly provided by People’s Hospital, Nankai University, Tianjin, China), respectively. The diluted DNA (500 μg/mL) and DOTAP-Cholesterol (8 mM) were then mixed at a 1:1 molar ratio and incubated at room temperature for 30 min to generate DOTAP-Cholesterol-encapsulated cuomIFNβ-pcDNA3.1 (termed IFNBCOL01) [39].

**In Vivo Tumor Mouse Model**. CT26 cells (2.5 × 10^5^ cells/mouse) were harvested and subcutaneously injected to the right flank of BALB/c mice. Ten days after tumor cell inoculation, tumor-bearing mice were randomly separated into two groups. DOTAP-Cholesterol (4 mM LNP, 100 μL/mouse) or IFNBCOL01 (25 μg DNA in 4 mM LNP, 100 μL/mouse) were i.v. injected into mice in two groups, respectively, once every 3 days, totaling four times. 

**CD11b^+^ Myeloid Cell Isolation.** Total tumor tissues were digested in a tissue digestion buffer (collagenase at 1 mg/mL, BS163, Biosharp, Hefei, China; hyaluronidase at 0.1 mg/mL, BS171, Biosharp, Hefei, China and DNase I at 30 U/mL, D8071, Solabrio, Beijing, China) at room temperature for approximately 40 min with agitation. The digests were then grinded and passed through a 70 µm cell strainer (251200, Sorfa, Huzhou, China). The cells were lysed with red cell lysis buffer for 5 min in room temperature and resuspended in PBS with 1% BSA. The CD11b^+^ myeloid cells were then purified from the tumor cell suspension using a Mouse CD11b Selection Kit (Cat# 480110, BioLegend, San Diego, CA, USA) and separated by a magnetic stand.

**CRISPR-Based Gene Knockout.** psPAX2 (BR036, Fenghuishengwu, Changsha, China), pCMV-VSVG (BR081, Fenghuishengwu, Changsha, China) and lentiCRISPRv2 (Genscript, Piscataway, NJ, USA) plasmids containing scramble (GAAGACTTAGTCGAATGAT) or IFNAR1-specific (TCAGTTACACCATACGAATC) sgRNA-coding sequences were co-transfected into HEK293T cells with Lipofectamine (Cat# T101-01, Vazyme, Nanjing, China). Virus particles in cell culture supernatants were collected and used to infect RAW264.7 cells. Stable cell lines were established by puromycin (Cat# BS111, Biosharp, Hefei, China) selection and then analyzed by flow cytometry for IFNAR1 expression verification.

**Western Blotting Analysis.** Cells were lysed in total lysis buffer on ice for one hour and measured for protein concentration using a Bradford Kit (Cat# MA0079, Meilunbio, Dalian, China), and cell lysates were separated in SDS-polyacrylamide gels (12% separation gel, 5% stacking gel) and transferred to PVDF membranes (Millipore, Burlington, MA, USA). The membranes were blotted with antibodies and developed by ECL. The antibodies are listed in Table S1. 

**Flow Cytometry Analysis.** For cell surface proteins, cells were harvested, centrifuged and washed in PBS. The cell pellets were then resuspended in 100 µL PBS and stained with fluorescent dye-conjugated antibodies. The suspension was then washed with PBS and resuspended in 300 µL PBS. Stained cells were analyzed using flow cytometry (BD FACS Verse). FlowJo v10 software (BD Biosciences, San Diego, CA, USA) was used to analyze data files. All antibodies were purchased from Biolegend and listed in Table S1.

**Chromatin Immunoprecipitation (ChIP) Assay.** ChIP assays were carried out according to the protocol of the Chromatin Immunoprecipitation Assay Kit (Cat# 17-295, Merck, NJ, USA). To sum up, cells (1 × 10^6^ cells) were harvested and cross-linked using 1% formaldehyde (Cat# F809702, Macklin, Shanghai, China). After a wash, the cells were resuspended in SDS lysis buffer and sonicated before centrifuge. The supernatant fraction was diluted ten fold by the ChIP dilution buffer (save 5% diluent as input). The immunoprecipitating antibody was added into the supernatant and incubated in 4 °C overnight with rotation. The next day, the protein A agarose was added into cell supernatant and incubated in 4 °C for one hour with rotation. The agarose was collected by centrifuge, washed in a low-salt immune complex wash buffer, high-salt immune complex wash buffer, LiCl immune complex wash buffer and TE buffer sequentially, and then eluted by 500 µL elution buffer. The eluants were recovered using phenol/chloroform/isoamyl alcohol (Cat# P59330, Acmec, Shanghai, China). The *irf2* and *cd279* promoter DNA was detected by quantitative PCR with promoter-DNA-specific primers as listed in Table S2.

**Gene Expression Analysis**. qPCR and regular PCR analysis were performed as previously described [40]. The sequences of primers are listed in Table S2.

**Statistical Analysis**. All statistical analyses were performed using a two-sided Student *t* test or a Dunnett’s test using the GraphPad Prism program (GraphPad Software, Inc., San Diego, CA, USA). *p* < 0.05 is considered statistically significant.

## 3. Results

### 3.1. PDCD1 Expression has a Positive Correlation with IFN-I Expression in Myeloid Cells in Human Colorectal Cancer

**Figure 1:**
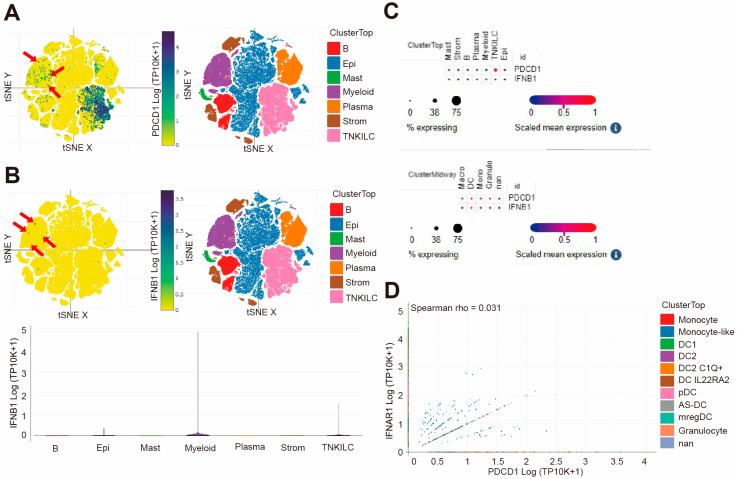
***PDCD1* and *IFNΒ1* expression profiles in human colorectal cancer patients.** (**A**) Shown are the UMAP of major cell subpopulations (**right panel**) and the *PDCD1* expression level (**left panel**) in the indicated cell subpopulations in human colorectal cancer. The red arrows show the *PDCD1* expression in myeloid cells. (**B**) Shown are the *IFNB1* expression level (**left panel**) in the indicated cell subpopulations and the UMAP of major cell subpopulations (**right panel**) in human colorectal cancer. The violin plot of *IFNB1* expression levels in the indicated major cell subpopulations is shown at the bottom (**left panel**). The red arrows show the *IFNB1* expression in myeloid cells. (**C**) Shown is the correlation between *IFNB1* and *PDCD1* in the cell subpopulations (**top panel**), as in A, and in the myeloid cell subpopulations (**bottom panel**). (**D**) Shown are correlations between *PDCD1* and *IFNAR1* in the myeloid cell subpopulations as shown in B.

To illustrate the correlation of *PDCD1* and *IFN-I* in human cancer patients, we analyzed the cellular source of *PDCD1* in colorectal cancer patients. We downloaded single cell RNA-seq datasets (GSE178341) [37] and analyzed *PDCD1* and *IFN-I* expression profiles in human colorectal tumors. Colorectal tumor resident cells were annotated (Figure 1A,B). Cellular subtype analysis revealed that *PDCD1* is primarily expressed in T, NK and ILCs (innate lymphocytes) (Figure 1A; combined cells “T, NK and ILCs” are indicated in Figure 1A as “TNKILC”), while *IFNB1* is primarily expressed in myeloid cells (Figure 1B) in human colorectal carcinoma. In contrast to *IFNB1*, *IFNA* expression is too weak to be detected in human colon carcinoma. Correlation analysis identified there is no correlations between *PDCD1* expression level and the *IFNB1* expression level in T, NK or ILCs (Figure 1C), but positive correlations do exist in myeloid cells subtypes (Figure 1C). Furthermore, *PDCD1* expression is positively correlated with *IFNAR1* expression in myeloid cells (Figure 1D). 

### 3.2. Myeloid Cell Intrinsic IFN-I Controls PD-1 Expression In Vitro

**Figure 2:**
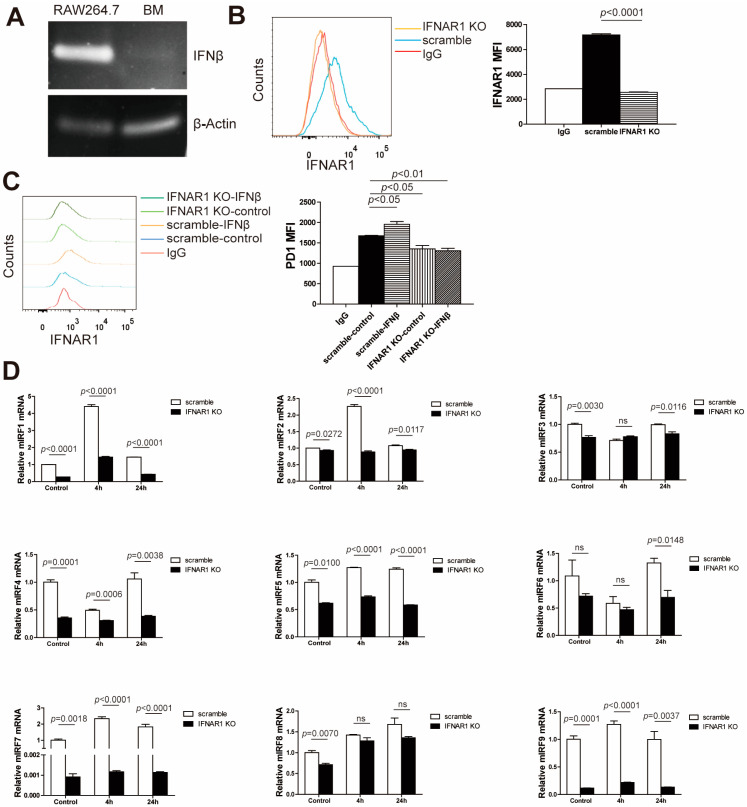
**Myeloid cell intrinsic IFN-I controls PD-1 expression in vitro.** (**A**) Total RNA was prepared from RAW264.7 and bone marrow-derived MDSCs, and analyzed for basal IFNβ mRNA expression by RT-PCR with β-actin as an internal control. (**B**) RAW264.7 scramble and RAW264.7 IFNAR1 KO cells were cultured in vitro for 24 h. Cells were stained with anti-IFNAR1 mAb and analyzed by flow cytometry with IgG as a negative control. The MFI (mean fluorescence intensity) of IFNAR1 was quantified and presented in the right panel. Column: mean; Bar: SEM. (**C**) RAW264.7 scramble and RAW264.7 IFNAR1 KO cells were treated with IFNβ (100 ng/mL) for 24 h. Cells were stained with anti-PD-1 mAb and analyzed using flow cytometry. Data were analyzed with Dunnett’s test. (**D**) Total RNA was extracted from RAW264.7 scramble and RAW264.7 IFNAR1 KO cells treated with IFNβ for 4 h and 24 h, respectively, and analyzed using qPCR for IRF1-9 expression. Shown is one representative result of three independent experiments, and the error bar is the mean of the triplicates for one experiment. Data were analyzed with the Student *t* test. ns: no significance.

To validate the above findings in mouse cells, we made use of mouse macrophage cell line RAW264.7 and bone marrow-derived MDSCs to model human myeloid cells. We first analyzed the basal level of IFNβ in these two cell lines. Although bone marrow-derived MDSCs produced much lower IFNβ levels compared with RAW264.7 cells (Figure 2A), both RAW264.7 cells and bone marrow-derived MDSCs secreted IFNβ. IFN-Is triggered signals through a dimeric IFNAR1/IFNAR2 receptor [41]. Additionally, it has been well established that autocrine IFN-I regulates PD-L1 expression through IFNAR1 in MDSCs [42]. To determine whether autocrine IFN-I directly regulates PD-1 expression in myeloid cells, *Ifnar1* was knocked out in RAW264.7 cells by the CRISPR technique. Flow analysis verified that *Ifnar1* had been efficiently knocked out (Figure 2B). The deletion of *Ifnar1* not only dramatically reduced the basal expression level of PD-1, but also significantly abrogated IFNβ-induced PD-1 upregulation in RAW264.7 cells (Figure 2C). These findings indicate that IFNβ regulates PD-1 expression in myeloid cells at least in part through an autocrine manner. 

IRFs (interferon regulatory factors) are transcription factors which are directly stimulated by JAK-STAT signaling pathways and induce different cellular responses by binding to the promoter regions of ISGs (interferon-stimulated genes) including PD-1 [41,43,44]. Our above observation that knocking out *Ifnar1* leads to downregulation of PD-1 suggests that IFN-I may modulate PD-1 expression through IRF regulation. To test this hypothesis, we analyzed the expression of IRF1-9. As expected, the deletion of *Ifnar1* significantly reversed IFNβ-induced IRF elevation in all IRF subtypes, especially IRF1, 2, 5, 7 and 9 (Figure 2D).

### 3.3. Extrinsic IFN-I Controls STATs-IRFs-PD-1 Expression in Myeloid Cells In Vitro

**Figure 3:**
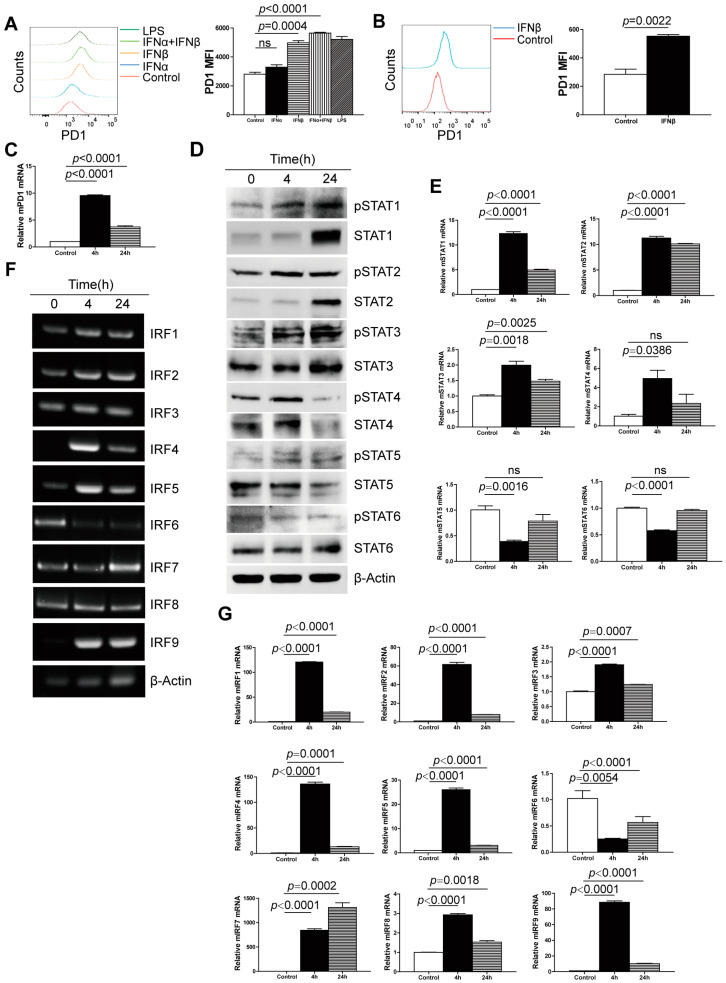
**Exogenous IFNβ activates STATs and IRFs to induce PD-1 expression in myeloid cells in vitro.** (**A**) RAW264.7 cells were treated with recombinant IFNα (100 ng/mL), IFNβ (100 ng/mL) and IFNα and IFNβ, respectively, for 24 h. LPS (2 ug/mL) is used as the positive control. Cells were stained with anti-PD-1 mAb and analyzed by flow cytometry. (**B**) The indicated RAW264.7 cells were treated with IFNβ alone for 24 h and then stained with anti-PD-1 mAb to be analyzed by flow cytometry. (**C**) Total RNA was isolated from RAW264.7 cells treated with IFNβ for 4 h and 24 h, respectively, and analyzed for the expression of PD-1 by qPCR. (**D**) RAW264.7 cells were treated with IFNβ for 4 h and 24 h, respectively, and lysed for total protein, then analyzed by Western blotting for the indicated STATs. β-actin is used as normalization control. (**E**) Total RNA was prepared from RAW264.7 cells treated with IFNβ for 4 h and 24 h, respectively, and analyzed for STAT1-6 expression using qPCR with β-actin as an internal normalization control. ns: no significance. (**F**,**G**) Total RNA was prepared from RAW264.7 cells treated with IFNβ for 4 h and 24 h, respectively, and analyzed for IRF1-9 expression by RT-PCR (**F**) and qPCR (**G**) with β-actin as internal normalization control. For RT-PCR, shown is one representative result of each of two independent experiments. For qPCR, shown is one representative result of each of three independent experiments, and the error bar is the mean of the triplicates for one experiment.

To strengthen the above findings, we took a complimentary approach by culturing RAW264.7 cells in the presence of recombinant IFNα and IFNβ proteins. Consistent with expectations, both IFNα and IFNβ treatment increased PD-1 expression. However, the PD-1 levels after IFNα treatment were slightly increased, but the increase was not statistically significant (Figure 3A). IFNβ significantly elevated PD-1 expression both at the protein level and RNA level (Figure 3B,C). It is well documented that IFNβ binds to IFNAR1 to stimulate ISG transcription through the STATs-IRFs axis. To determine which STATs are activated after IFNβ binding to IFNAR1, we analyzed six known STATs using different treatment times. IFNβ induced STAT1 and STAT3 phosphorylation in a time-dependent manner (Figure 3D). However, phosphorylated STAT2 and STAT4 increased first after 4 h and then decreased after 24 h (Figure 3D). In addition, both phosphorylated STAT5 and STAT6 protein levels were low and were not notably altered by IFNβ (Figure 3D). IFNβ also increased the protein level of non-phosphorylated STAT1 and STAT2 (Figure 3D). At the RNA level, all STATs were induced by IFNβ except for STAT5 and STAT6 (Figure 3E). Furthermore, we analyzed nine known IRFs using different treatment times by regular PCR and qPCR. IFNβ induced all IRFs except for IRF6 (Figure 3F,G), which may be regulated by other transcription repressors which are induced by IFNβ. Among the induced IRFs, IRF7 was stimulated by IFNβ time-dependently, while the other IRFs were stimulated by IFNβ after 4 h, but the inductive effect decreased after 24 h (Figure 3F,G). 

### 3.4. IFNβ Controls STATs-IRFs-PD-1 Expression in Myeloid Cells In Vivo

**Figure 4:**
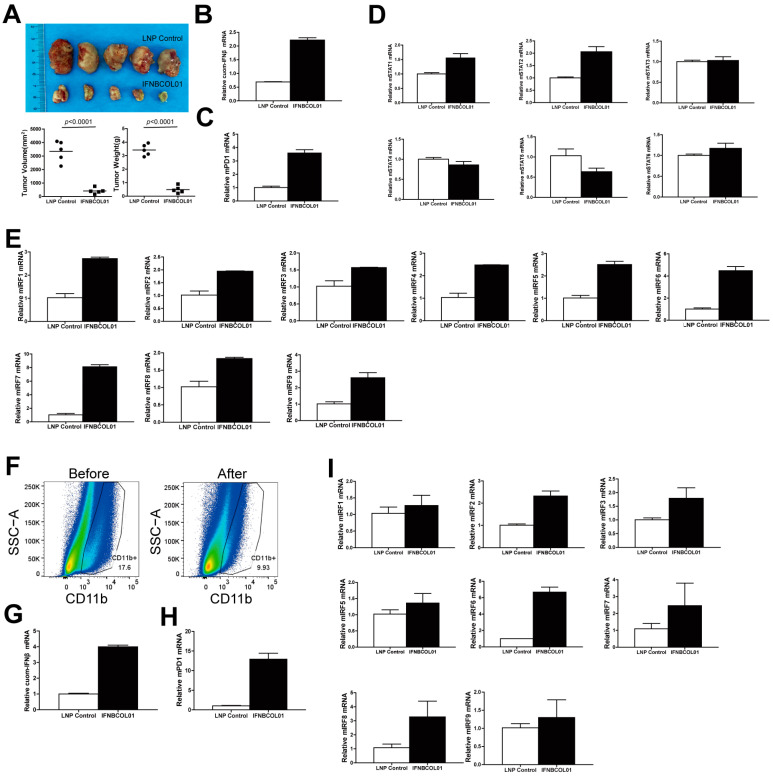
Overexpression of IFNβ in the tumor microenvironment activates STATs and IRFs to stimulate PD-1 expression in myeloid cells in vivo. (**A**) CT26 cells (2.5 × 10^5^ cells/mouse) were subcutaneously injected in mice to establish in vivo tumor models. The tumor-bearing mice were then intravenously treated with LNP (n = 5) and IFNBCOL01 (n = 5) 10 days after tumor inoculation, once every 3 days, totaling 4 times. Shown are the tumor images (top panel). The tumor volume and weight were quantified and shown in the bottom panels with LNP as a control. (**B**–**E**) Total RNA was prepared from the total tumor tissues, as shown in A, and analyzed for the expression of the indicated genes using qPCR with β-actin as an internal control. (**F**) Tumor tissues, as shown in A, were digested into single cells and used CD11b positive selection beads to isolate CD11b^+^ myeloid cells. Tumor tissues before and after isolation were stained by anti-CD11b mAb and analyzed by flow cytometry. Shown is the percentage of CD11b^+^ myeloid cells before isolation (**left panel**) and CD11b^+^ myeloid cells in supernatant after isolation (**right panel**). (**G**–**I**) Total RNA was prepared from CD11b^+^ myeloid cells as shown in E and analyzed for the mRNA level of the indicated genes using qPCR with β-actin as an internal normalization control. For qPCR, shown is one representative result of two independent experiments, and the error bar is the mean of the triplicates for one experiment. CT26 cells (2.5 × 10^5^ cells/mouse) were harvested and subcutaneously injected into the right flank of BALB/c mice. Ten days after tumor cell inoculation, tumor-bearing mice were randomly separated into two groups. DOTAP-Cholesterol (4 mM LNP, 100 μL/mouse) or IFNBCOL01 (25 μg DNA in 4 mM LNP, 100 μL/mouse) were i.v. injected into mice in the two groups, respectively, once every 3 days for a total of 4 times.

Our above findings indicated that intrinsic IFNAR1 knocked out abolished IFNβ-induced PD-1 and IRF upregulation in myeloid cells. On the other hand, extrinsic IFNβ treatment elevated PD-1 expression through the STATs-IRFs axis in myeloid cells in vitro. To determine whether the above findings can be translated to colon tumor growth inhibition and PD-1 expression regulation in vivo, we subcutaneously injected CT26 tumor cells to syngeneic mice to establish an in vivo colon tumor model. The tumor-bearing mice were then intravenously injected with IFNΒCOL01 (a lipid nanoparticle-encapsulated IFNβ-encoding plasmid) [39]. IFNΒCOL01 effectively delivered cuomIFNβ plasmid DNA to the tumor microenvironment (Figure S1) and cuomIFNβ was highly expressed in total tumor tissue RNA levels (Figure 4B). IFNΒCOL01 therapy dramatically repressed the established tumor growth in mice (Figure 4A) and upregulated PD-1 expression in the tumor microenvironment (Figure 4C). Our previous findings demonstrated that the overexpression of IFNβ in a tumor microenvironment leads to the enhanced expression of Gzmb (granzyme B) and MHC I (major histocompatibility complex I), resulting in effective tumor suppression in vivo [39]. This will be further discussed in the Discussion section. The analysis of IRFs and STATs in total tumor tissues showed that IFNΒCOL01 therapy led to elevated STAT1, 2 and 6 levels (Figure 4D) and expression of all IRFs (Figure 4E).

To demonstrate the effect of IFNΒCOL01 in tumor-infiltrating myeloid cells, we isolated CD11b^+^ cells from total tumor tissues by CD11b selection beads. The isolation efficiency is shown in Figure 4F. Consistent with the results in total tumor tissues, cuomIFNβ was highly expressed in the RNA level in IFNBCOL01-treated tumor-infiltrating CD11b^+^ cells (Figure 4G). Accordingly, IFNΒCOL01 augmented PD-1 (Figure 4H) and IRFs expression (except IRF4) (Figure 4I) in CD11b^+^ cells. Immune cell profiles analysis revealed that IFNΒCOL01 increased CXCL10 and MHC-I expression in CD11b^+^ cells (Figure S2), which may contribute to tumor growth inhibition.

### 3.5. IFNβ Elevates PD-1 Expression through pSTAT1-IRF2 Axis in Myeloid Cells

**Figure 5:**
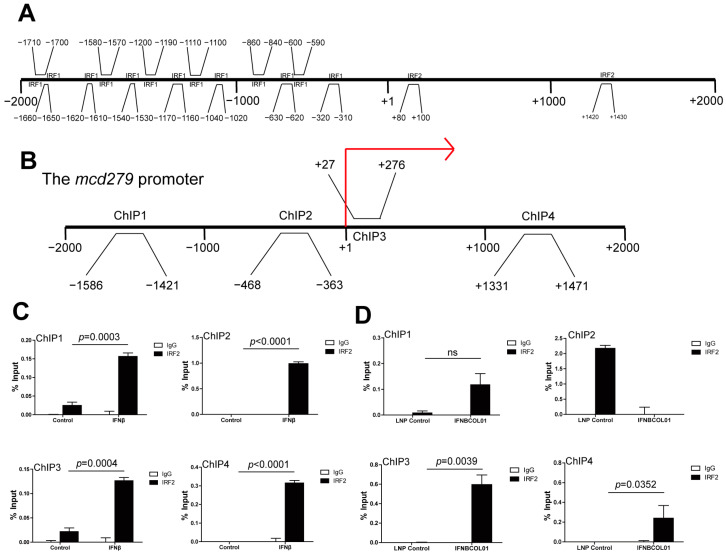
**IRF2 binds to *cd279* promoter region in myeloid cells.** (**A**) The mouse *cd279* promoter DNA sequence (−2000 to +2000 relative to the *cd279* transcription start site) was exported from the mouse genomic database and analyzed for potential IRF-binding elements using the *PROMO* database. Shown is the mouse *cd279* promoter structure with predicted binding locations of IRF1 and IRF2 to the *cd279* promoter region. (**B**) Four pairs of primers spanning from −2000 to +2000 relative to the mouse *cd279* promoter region are shown. The red arrow represents the transcription start site. (**C**) RAW264.7 cells were treated with IFNβ for 24 h. Chromatin was then prepared and analyzed using ChIP with an IRF2-specific antibody. The immunoprecipitated chromatin fragments were then analyzed by qPCR with primers shown in (**B**). (**D**) Chromatin was prepared from IFNΒCOL01-treated CD11b^+^ tumor-infiltrating myeloid cells shown in Figure 4F and analyzed using ChIP with a IRF2-specific antibody as in (**C**). The immunoprecipitated chromatin fragments were then analyzed with qPCR as in (**C**). For ChIP analysis, shown is one representative result of two independent experiments, and the error bar is the mean of the triplicates for one experiment. ns: no significance.

To reveal the specific STATs-IRFs axis used by IFNβ to modulate PD-1 expression in myeloid cells, we exploited *PROMO* software to make transcription factor predictions at the mouse *cd279* promoter region. As shown in Figure 5A, among the nine known IRFs, only IRF1 and IRF2 were predicted to bind at the *cd279* (the gene encodes the murine PD-1) promoter region (−2000 to +2000 bp). IRF2 is known to antagonize IRF1 by competing for binding to the same promoter elements of IFN-I- and IFN-II-inducible genes [45] and by inhibiting the nuclear translocation of IRF1 [46]. Furthermore, IRF2 is reported to be associated with T cell exhaustion and function [47]. Therefore, we analyzed the protein level of IRF2 at different times. As expected, IRF2 was induced by IFNβ time-dependently as IFNγ (Figure S3).

To validate whether IRF2binds to the mouse *cd279* promoter, we treated RAW264.7 cells with recombinant IFNβ and treated CT26 tumor-bearing mice with IFNBCOL01, then performed ChIP analysis using an IRF2-specific antibody and PCR primers that covered the promoters approximately from −2000 to +2000 relative to the transcription start site of the mouse *cd279* (Figure 5B). Analysis of the mouse *cd279* promoter regions by ChIP illustrated that after IFNβ and IFNΒCOL01 treatment, enrichment of *irf2* increased in the promoter regions of the *cd279* gene in −2000 to +2000 regions in RAW264.7 cells (Figure 5C) and in −2000 to 0, +1000 to +2000 regions in tumor-infiltrating CD11b^+^ cells (Figure 5D).

**Figure 6:**
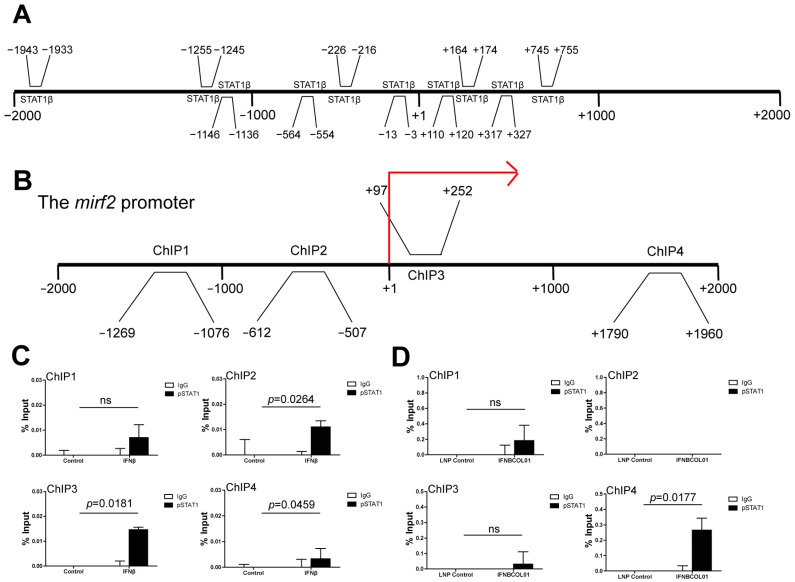
**pSTAT1 binds to the *irf2* promoter region in myeloid cells.** (**A**) The mouse *irf2* promoter DNA sequence (=−2000 to +2000 relative to *irf2* transcription start site) was exported from the mouse genomic database and analyzed for potential STAT-binding elements using the *PROMO* database. Shown is the *irf2* structure with predicted binding locations of STAT1β to the *irf2* promoter region. (**B**) Four pairs of primers spanning from −2000 to +2000 relative to the *irf2* promoter region are shown. The red arrow represents the transcription start site. (**C**) Chromatin was prepared from RAW264.7 cells treated with IFNβ for 24 h and analyzed using ChIP with a pSTAT1-specific antibody. The immunoprecipitated chromatin fragments were then analyzed using qPCR with primers shown in (**B**). (**D**) Chromatin was prepared from IFNΒCOL01-treated CD11b^+^ tumor-infiltrating myeloid cells shown in Figure 4F and analyzed using ChIP with a pSTAT1-specific antibody, as in (**C**). The immunoprecipitated chromatin fragments were then analyzed using qPCR, as in (**C**). For ChIP analysis, shown is one representative result of two independent experiments, and the error bar is the mean of the triplicates for one experiment. ns: no significance.

To further determine which specific STAT binds to the *irf2* promoter region, we used *PROMO* software to perform transcription factor prediction at the mouse *irf2* promoter region. As shown in Figure 6A, among six known STATs, only STAT1 was predicted to bind at the *irf2* promoter region (−2000 to +2000 bp). Consistent with the prediction, IFNAR1 deficiency dramatically attenuated the induction of pSTAT1 by IFNβ (Figure S4). To validate whether pSTAT1 binds to the mouse *irf2* promoter, we treated RAW264.7 cells with recombinant IFNβ and treated CT26 tumor-bearing mice with IFNBCOL01, then performed ChIP analysis using a pSTAT1 specific antibody and PCR primers that covered the promoters approximately from −2000 to +2000 relative to the transcription start site of the mouse *irf2* (Figure 6B). Analyses of mouse *irf2* promoter regions by ChIP demonstrated that after IFNβ and IFNBCOL01 treatment, enrichment of pSTAT1 increased in the promoter regions of the *irf2* gene in −2000 to +2000 regions in RAW264.7 cells (Figure 6C), and in −2000 to 0, +1000 to +2000 regions in tumor-infiltrating CD11b^+^ cells (Figure 6D).

### 3.6. IFNβ Regulates PD-1 Expression through pSTAT1-IRF2 Axis in Bone Marrow-Derived MDSCs

**Figure 7:**
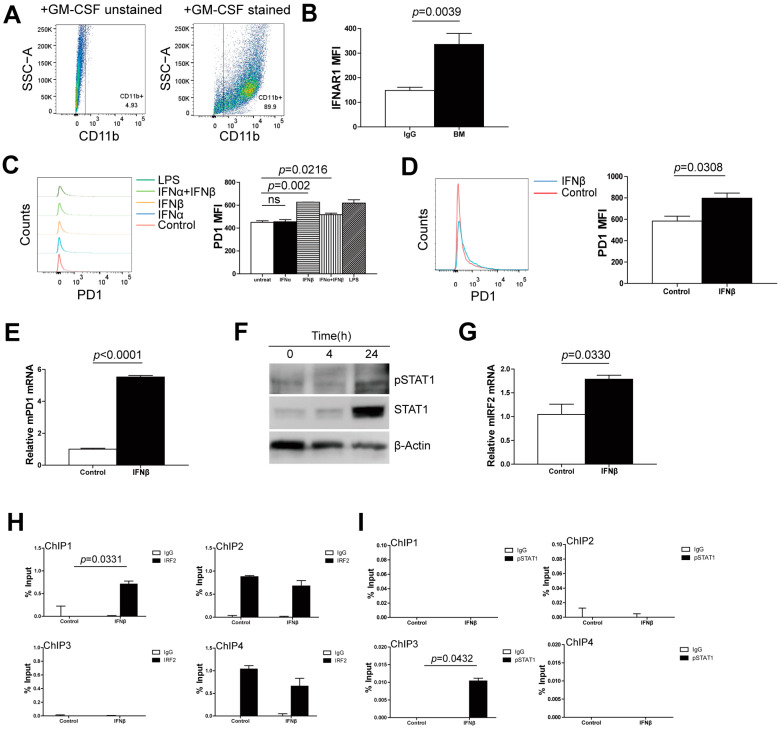
**IFNβ-STAT1-IRF2 axis upregulates PD-1 expression in bone marrow-derived MDSCs.** (**A**) Bone marrow cells were prepared from BALB/c WT mice and treated with GM-CSF (2 ng/mL) for 7 days. Cells were then stained with anti-CD11b mAb and analyzed using flow cytometry. The percentage of CD11b^+^ myeloid cells after GM-CSF treatment (right pane) is shown. (**B**) Bone marrow-derived MDSCs were cultured for 24 h and then stained with anti-IFNAR1 mAb and analyzed by flow cytometry. (**C**) Bone marrow-derived MDSCs were treated with recombinant IFNα, IFNβ and IFNα+IFNβ, respectively, for 24 h. LPS was used as the positive control. Cells were then stained with anti-PD-1 mAb and analyzed by flow cytometry. ns: no significance. (**D**) The bone marrow-derived MDSCs were treated with IFNβ alone for 24 h, and then stained with anti-PD-1 mAb and analyzed by flow cytometry. (**E**) Total RNA was isolated from bone marrow-derived MDSCs treated with IFNβ for 4 h and 24 h, respectively, and analyzed for the mRNA level of PD-1 using qPCR with β-actin as the internal control. (**F**) Bone marrow-derived MDSCs were treated with IFNβ for 4 h and 24 h, respectively, and lysed for total protein, and then analyzed by Western blotting for STAT1 and pSTAT1. β-actin is used as the normalization control. (**G**) Total RNA was prepared from bone marrow-derived MDSCs treated with IFNβ for 4 h and 24 h, respectively, and analyzed for the expression of IRF2 using qPCR. (**H**,**I**) Chromatin was isolated from bone marrow-derived MDSCs treated with IFNβ for 24 h and analyzed by ChIP with an IRF2-specific antibody, (**H**) as in Figure 5C, and a pSTAT1-specific antibody, (**I**) as in Figure 6C. The immunoprecipitated chromatin fragments were then analyzed using qPCR with primers shown in Figure 5B and Figure 6B, respectively. For ChIP analysis, shown is one representative result of two independent experiments, and the error bar is the mean of the triplicates for one experiment.

Our above results showed that IFNβ elevated PD-1 expression through the pSTAT1-IRF2 axis in myeloid cells both in vitro and in vivo. To further validate this finding, we made use of another myeloid cell line: bone marrow-derived MDSCs. After GM-CSF induction, around 90% of the bone marrow cells were CD11b^+^ (Figure 7A). These bone marrow-derived MDSCs expressed IFNAR1 (Figure 7B), which suggested that they have the ability to respond to IFN-I through IFNAR1. Just like with the RAW264.7 cells, we cultured bone marrow-derived MDSCs in the presence of recombinant IFNα and IFNβ protein. Only IFNβ treatment augmented PD-1 expression, while IFNα has no inductive effect (Figure 7C). IFNβ upregulated PD-1 expression both at the protein level and RNA level (Figure 7D,E). IFNβ also induced pSTAT1 and IRF2 expression in bone marrow-derived MDSCs (Figure 7F,G). 

To validate the binding of pSTAT1 to the *irf2* promoter and the binding of IRF2 to the *cd279* promoter in bone marrow-derived MDSCs, we treated bone marrow-derived MDSCs with recombinant IFNβ, then performed ChIP analysis. PCR primers that covered the promoters from −2000 to +2000 relative to the transcription start sites of mouse *cd279* and *irf2* are shown in Figure 5B and Figure 6B. The analysis of mouse *cd279* promoter regions by ChIP demonstrated that after IFNβ treatment, enrichment of IRF2 increased in the promoter region of the *cd279* gene in −2000 to −1000 region (Figure 7H), and enrichment of pSTAT1 was elevated in the promoter region of the *irf2* gene in the 0 to +1000 region (Figure 7I), respectively, in bone marrow-derived MDSCs.

### 3.7. IRF2 Expression Exhibits Positive Correlation with STAT1/PDCD1 Expression in Myeloid Cells in Human Colon Cancer

Our above findings indicated IFNβ-regulated PD-1 expression through the pSTAT1-IRF2 axis in three different types of myeloid cells (RAW264.7 cells, tumor-infiltrating CD11b^+^ cells and bone marrow-derived MDSCs). To illustrate the human cancer relevance of our above findings, we then analyzed the cellular source of IRF2 in colon cancer patients. We extracted scRNA-seq datasets (GSE178341) [37] and analyzed *IRF2* expression profiles in human colon tumors. Colon tumor-resident cells were annotated (Figure S5A). Cellular subtype analysis indicated that *IRF2* is primarily expressed in T, NK, B, stroma and myeloid cells in human colon carcinoma (Figure S5A).

Correlation analysis revealed positive correlations between *IRF2* and *STAT1* expression levels, *IRF2* and *PDCD1* expression levels and *PDCD1* and *STAT1* expression levels (Figure S5B–D). Moreover, *IRF2*, *STAT1* and *PDCD1* expression levels are positively correlated with each other in different subtypes of myeloid cells (Figure S5E).

## 4. Discussion

PD-1, associated with the expression of additional repressive receptors such as Tim-3, LAG-3 or TIGIT [48,49,50], is a co-inhibitory receptor of the CD28 family and functions as a negative regulator to provide crucial inhibitory signals that maintain immune homeostasis, control inflammation resolution, regulate host immune defense and mediate tolerance. PD-1 therefore functions as an immune checkpoint to prevent autoimmunity [1,5]. Nevertheless, under pathological conditions such as chronic viral infection and cancer, sustained PD-1 expression leads to chronic T cell activation to induce T cells into dysfunction and exhaustion. In cancer patients and tumor-bearing mice, tumor cells and tumor cell-induced myeloid cells express high levels of PD-L1 in response to IFNγ secreted by tumor-activated T cells. PD-L1 binds to PD-1 on T cells to induce T cell dysfunction and exhaustion to promote tumor immune evasion and resultant progression [51]. Tumor cells hijack the PD-1/PD-L1 immune checkpoint to dampen the anti-tumor immune response. T cell-expressed PD-1 therefore serves as a crucial target for tumor cell counterattack. The expression and regulation of PD-1 in T cells have been extensively studied and multiple layers of transcriptional regulatory mechanisms have been defined in the regulation of PD-1 expression in T cells, including different epigenetic and genetic programs adapted to either transient [52] or chronic antigenic stimulations [26,53,54]. However, it has been recently observed that myeloid cell PD-1 plays an essential role in the suppression of anti-tumor immunity [34,35,36]. Knocking out myeloid cell PD-1 leads to increased tumor reactive T cells and enhanced tumor growth control despite preserved PD-1 expression in the T cells of the tumor-bearing mice [36]. The revelation of this key PD-1 function in myeloid cells in tumor immune suppression and evasion suggests a critical role of myeloid cell PD-1 expression regulation in tumor immune evasion and the mechanism by which a tumor responds to ICI immunotherapy. In this study, we determined that IFNβ upregulates PD-1 expression and knocks out IFNAR1-suppressed PD-1 expression in myeloid cells, revealing that PD-1 expression is regulated by IFN-I/IFNβ at least in part through an autocrine mechanism in myeloid cells in vitro and in tumor-bearing mice in vivo. We also observed that the PD-1 expression level is also positively correlated with the expression levels of IFNB1 and IFNAR1 in the myeloid cells of human colon cancer patients. Therefore, PD-1 expression regulation by IFNβ is a physio-pathologically relevant mechanism. 

PD-1 is an inhibitory receptor that inhibits myeloid cell activation and differentiation to impair host anti-tumor immunity [34,35,36,55,56,57,58]. Although IFN-I has been shown to promote tumor stemness under certain cellular contexts [59,60], IFN-I is the main defense line against pathogen infection in the host immune surveillance system [61] and is also the master regulator of anti-tumor immunity [62,63,64,65,66]. It is also known that the engagement of myeloid cell PD-1 by tumor PD-L1 suppresses IFN-I expression and signaling [35]. The upregulation of PD-1 by IFNβ therefore makes no sense in terms of the opposite functions of PD-1 and IFNβ under physiological conditions. However, this phenomenon is not uncommon as it is also known that the expression of PD-L1, the immune-suppressive ligand for PD-1, is also upregulated by IFNγ [67], an essential cytokine for T cell function in host cancer immune surveillance [68]. This phenomenon is believed to be an adaptive immune resistance process where the cancer changes its phenotype in response to activated T cell attack and thereby evading it [69,70]. We therefore propose that the upregulation of PD-1 by IFNβ in myeloid cells is an indicator of myeloid cell activation and another adaptive immune resistance process in cancer. Myeloid cells respond to cancer by activating IFN-I/IFNβ expression to regulate an anti-tumor immune response, but at the same time, tumor cells may use PD-L1 to activate PD-1-intrinsic inhibitory signaling in myeloid cells to shut down IFN-I expression and therefore function to evade cancer. IFNBCOL01 therapy dramatically improved the expression of Gzmb and MHC I in the tumor microenvironment [39]. This tumor-suppression effect may overcome the tumor promotion effect caused by the shut-down of IFN-I signaling in myeloid cells, leading to overall tumor inhibition. 

IFN-I transduces signals through a dimeric IFNAR1/IFNAR2 receptor that activates the kinases Jak1 and Tyk2 to initiate STAT phosphorylation to activate the expression of IFN-I-stimulated genes (ISGs) [41]. In this study, we determined that IFNβ activates STAT1, and activated STAT1 directly binds to the *irf2* promoter to activate IRF2 expression. Furthermore, IRF2 directly binds to the *cd279* promoter to activate PD-1 expression in myeloid cells. A positive correlation between STAT1, IRF2 and PD-1 is observed in the myeloid cells in human colon cancer patients. It has been well documented that IRF2 expression by CD8^+^ tumor-infiltrating lymphocytes drives T cell exhaustion, and *irf2*^−/−^ CD8^+^ T cells expressed lower levels of inhibitory receptors including PD-1 as compared with WT CD8^+^ T cells [47,71]. However, how the IFNβ/STAT1/IRF2 pathway directly regulates PD-1 expression in myeloid cells remains to be determined. Nevertheless, in this research, we determined that the IFNβ/STAT1/IRF2 axis regulates PD-1 expression in myeloid cells.

## 5. Conclusions

PD-1 is a co-repressive receptor of T cells and a key target in human cancer immunotherapy. Emerging experiment data revealed a key role of the intrinsic PD-1 pathway in myeloid cells. The regulation of PD-1 expression in T cells has been extensively studied, but the regulation of PD-1 expression in myeloid cells is incompletely understood. We determined that the expression level of PDCD1 is positively correlated with the levels of IFNB1 and IFNAR1 in myeloid cells in human colorectal cancer. Recombinant IFNβ protein treatment elevated PD-1 expression in mouse myeloid cells in vitro. Furthermore, deletion of IFNAR1 reversed the inductive effect of IFNβ on PD-1 expression in myeloid cells in vitro. The forced expression of IFNβ in tumor-bearing mice led to elevated PD-1 expression in tumor-infiltrating myeloid cells in vivo. At the molecular level, we determined that IFNβ activates STAT1. The activated STAT1 (pSTAT1) binds to the *irf2* promoter region to elevate IRF2 expression. IRF2 then binds to the *Pdcd1* promoter region to increase PD-1 expression. In human colon cancer patients, the expression levels of STAT1, IRF2 and PDCD1 are positively correlated in tumor-infiltrating myeloid cells. Our findings thus determined that IFNβ activates PD-1 expression via the pSTAT1-IRF2 pathway in myeloid cells, which provides the molecular mechanism for targeting myeloid cell PD-1 expression in cancer immunotherapy.

## Data Availability

Publicly available datasets were analyzed in this study. The datasets are described in the method and results section.

## Supplementary Materials

The following supporting information can be downloaded at: https://www.mdpi.com/article/10.3390/cells13131163/s1, Figure S1: IFNBCOL01 delivery of cuomIFNβ to tumor site; Figure S2: Immune cell profiles in tumor-infiltrating CD11b^+^ cells after IFNBCOL01 therapy; Figure S3: IFNβ induced IRF2 expression; Figure S4: IFNAR1 deletion diminished the induction of pSTAT1 by IFNβ; Figure S5: Correlation between STAT1, IRF2 and PDCD1 in human colon cancer; Figure S6: Original Western blots (related to Figure 3D, Figure S3, Figure 7F and Figure S4); Table S1: Antibodies; Table S2: Primer sequences.
