# Supplementary material for: Type I Interferon Activates PD-1 Expression through Activation of the STAT1-IRF2 Pathway in Myeloid Cells
Source: Cells. 2024 Jul 8;13(13):1163. doi: 10.3390/cells13131163 (PMC11240780; doi:10.3390/cells13131163)
Supplement: Supplementary file 1 [file cells-13-01163-s001.zip › cells-3014398-supplementary.pdf]

Supplemental Data

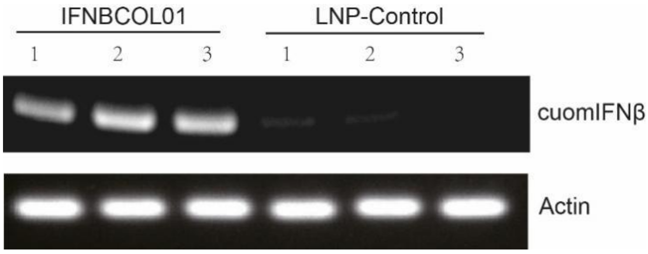

Figure S1. IFNBCOL01 delivery of cuomIFNβ to tumor site. Genomic DNA is extracted from total tumor tissues from LNP-Control and IFNBCOL01 treated tumor tissues from tumor-bearing mice.

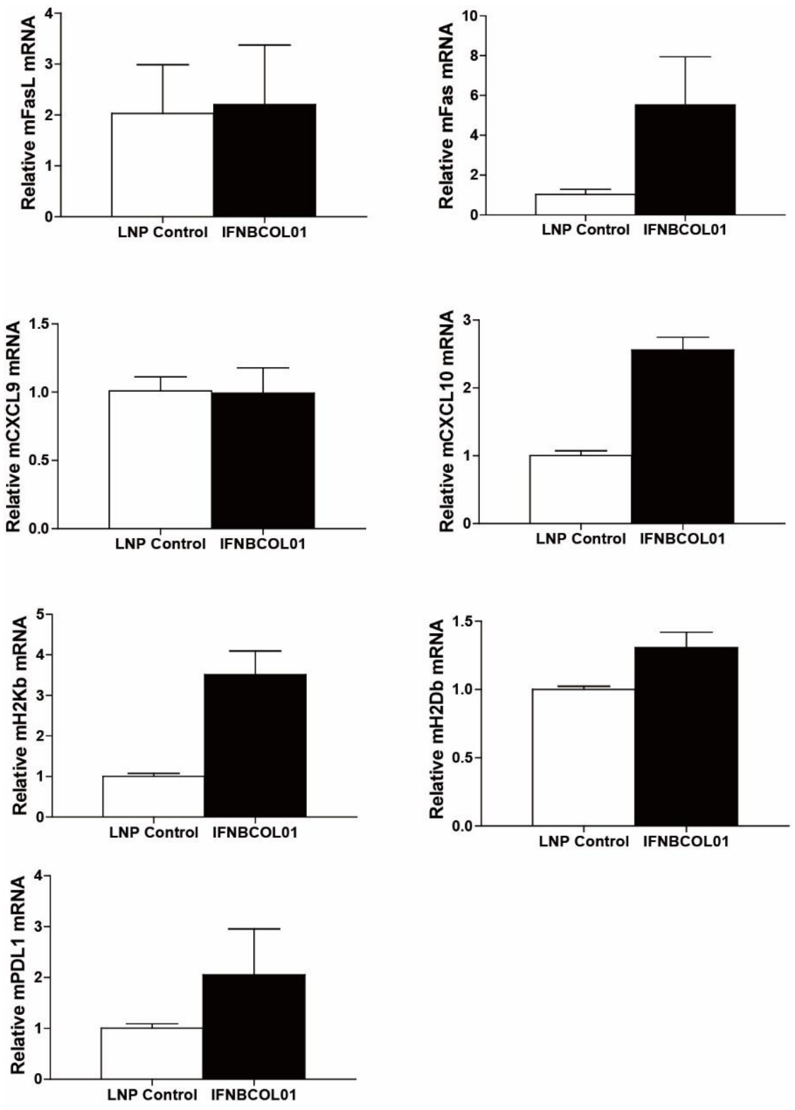

Figure S2. Immune cell profiles in tumor-infiltrating CD11b$^+$ cells after IFNBCOL01 therapy. Total RNA was prepared from the CD11b$^+$ cells isolated from the LNP Control and IFNBCOL01-treated tumor tissues, respectively as shown in Figure 4A and analyzed for the expression of the indicated genes by qPCR with β-actin

as internal control. Shown is one representative result of two independent experiments, and the error bar is the mean of triplicates for one experiment. Column: mean, bar: SEM.

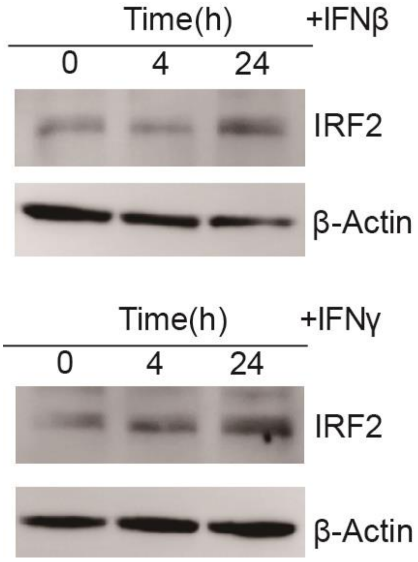

Figure S3. IFNβ induced IRF2 expression. RAW264.7 cells were treated with IFNβ (100ng/ml) and IFNγ (100ng/ml) for 4h and 24h, respectively, and lysed for total protein, then analyzed by Western blotting for IRF2. β-actin is used as normalization control.

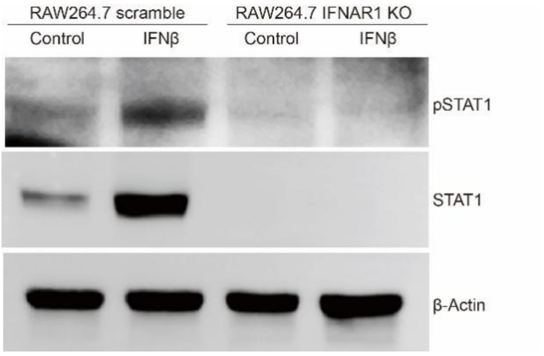

Figure S4. IFNAR1 deletion diminished the induction of pSTAT1 by IFNβ. RAW264.7 scramble and RAW264.7 IFNAR1 KO cells were treated with recombinant IFNβ for 24h, and lysed for total protein, followed by analysis by Western blotting for the expression of STAT1 and pSTAT1. β-actin is used as normalization control.

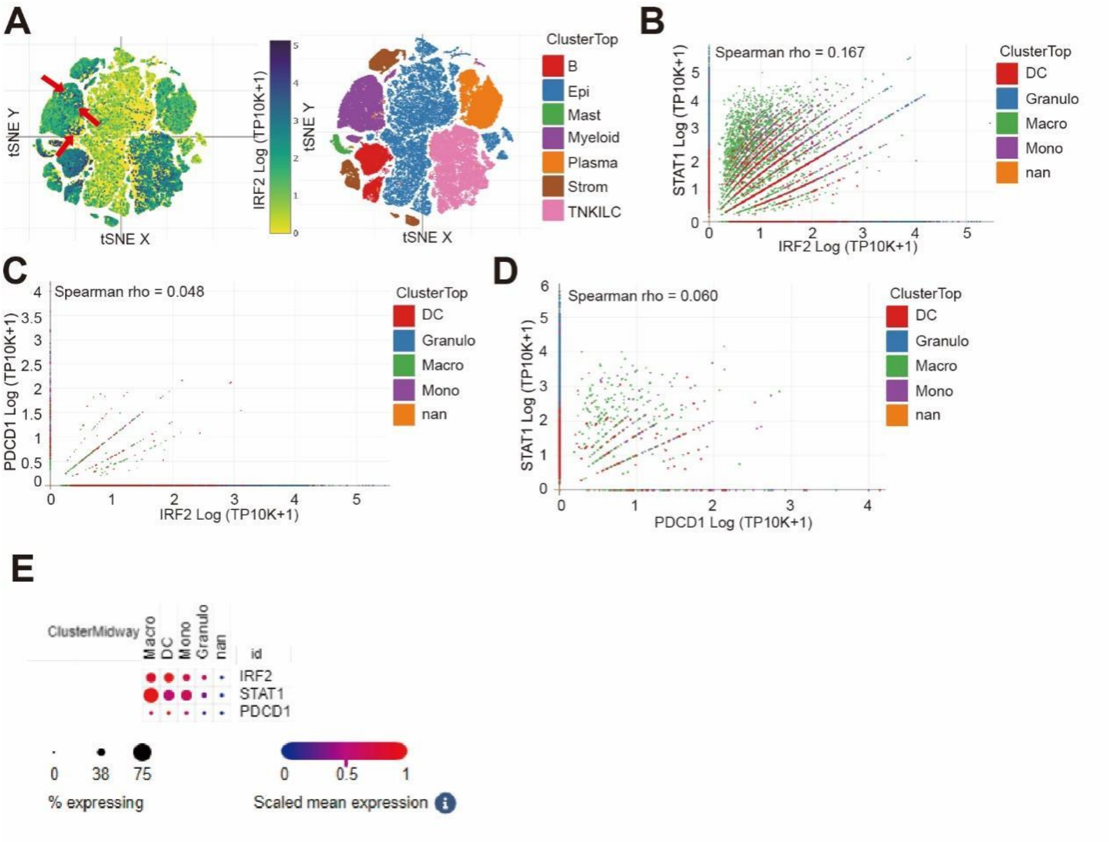

Figure S5. Correlation between *STAT1, IRF2* and *PDCD1* in human colon cancer patients. A. UMAP of major cell subpopulations (right panel) and *IRF2* expression level (left panel) in the indicated cell subpopulations in human colon cancer. **B to D.** Correlations between *IRF2* and *STAT1*(B), *IRF2* and *PDCD1* (C), *STAT1* and *PDCD1* (D), respectively, in the myeloid subpopulations. **E.** Correlations between *IRF2*, *STAT1* and *PDCD1* in the myeloid cell subpopulations.

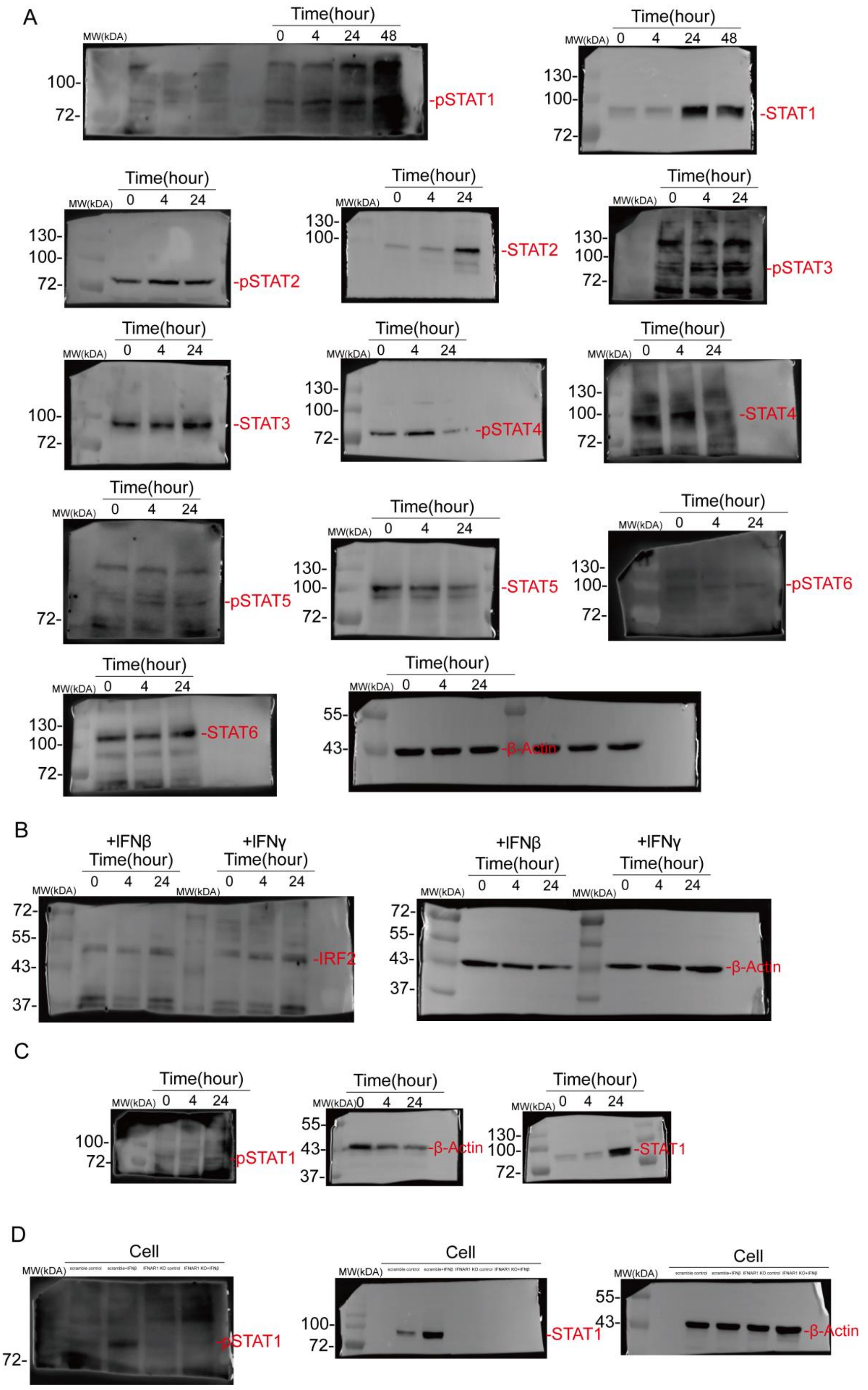

Figure S6. Original Western blots (related to Figures 3D, S3, 7F and S4)

Table S1. Antibodies

Name	Company	Catlog#	Application
Stat1(D1K9Y) Rabbit mAb	Cell Signaling	14994	WB
Stat2(D9J7L) Rabbit mAb	Cell Signaling	72604	WB
Stat3(D1B2J) Rabbit mAb	Cell Signaling	30835	WB
Stat4(C46B10) Rabbit mAb	Cell Signaling	2653	WB
Stat5(D2O6Y) Rabbit mAb	Cell Signaling	94205	WB
Stat6(D3H4) Rabbit mAb	Cell Signaling	5397	WB
Phospho-Stat1(Tyr701) (D4A7) Rabbit mAb	Cell Signaling	7649	WB, ChIP
Rabbit Anti-Phospho-Stat2(Tyr690) Polyclonal Antibody	Bioss	bs-3428R	WB
Phospho-Stat3(Tyr705) (D3A7) XP Rabbit mAb	Cell Signaling	9145	WB
Stat4(Phospho-Tyr693) Antibody	SAB	11047	WB
Phospho-Stat5(Tyr694) (D47E7) XP Rabbit mAb	Cell Signaling	4322	WB
Stat6(Phospho-Tyr641) Antibody	SAB	11050	WB
β-Actin Rabbit mAb	SAB	52901	WB
FITC anti-mouse CD279 (PD1)	Biolegend	135214	Flow Cytometry
FITC anti-mouse/human CD11b	Biolegend	101206	Flow Cytometry
APC anti-mouse IFNAR-1	Biolegend	127313	Flow Cytometry
IRF2(G-10) X	Santa cruz	sc-374327	WB, ChIP
Normal mouse IgG	Santa cruz	sc-2025	ChIP
Rabbit IgG control Polyclonal antibody	proteintech	30000-0-AP	ChIP

Table S2. Primer sequences

Gene	Forward primer	Reverse primer
mPD-1	CCGCCTTCTGTAATGGTTTGAG	CGATTTTTGCCTTGGGGTGC
mPD-L1	ATTGCTCCTTGACTGCTGGCTG	TTCTGGGTTCCTCCTCCTTTCC
mFas	ATGCTGTGGATCTGGGCT	TCACTCCAGACATTGTCC
mFasL	CTTGGGCTCCTCCAGGGTCAGT	TCTCCTCCATTAGCACCAGATCC
mCXCL9	TCATTGCTACACTGAAGAACGGAG	ACGACGACGACTTTGGGGTG
mCXCL10	TCTCTCCATCACTCCCCTTTACC	CTTGCTTCGGCAGTTACTTTTGTC
mH2Kb	TCTTGTACCTGTCCTTCCCAGA	CTCTGCCCTTTCCTACCTGTG
mH2Db	AGCAGAGTTTCCGAGTGGAC	TTCAGGTCTTCGTTCAGGGC
mIFNβ	CTGCGTTCCTGCTGTGCTTC	TCTTCTCCGTCATCTCCATAGGG
mcuom-IFNβ	CCACGCTGCCTTCTTGTTGTG	CCGCTCCTCTTGTTTTTCTTCC
mIRF1	TAACTCCAGCACTGTCACCGTG	TATGCCTATCCCAATGTCCCC
mIRF2	GCTGCCCTTATCCGAACGAC	GCGTAGGAAGACACAGGAGAAATC
mIRF3	CACGCTACACTCTGTGGTTCTGC	GCTGGCTGTTGGAGATGTGC
mIRF4	CCACGGACACACCTATGATGTTAG	AGAATGACGGAGGGAGCGG
mIRF5	CCATCCGTCTGTGCCAGTGTAAG	AACATCTCCAGCAGCAACCG
mIRF6	TGGCTACACAGAGATTCCAAACG	TGGGGGATGTCACACACTTGATAG
mIRF7	CCCCAGCAGTAAGAACTTCAGAGC	TAGTGTGGTGACCCTTGCCGCCTC
mIRF8	GAAGACCCATTCACTGGCATCTC	CAAACTAACTCCTTTTCAGCGGC
mIRF9	CATCCCCATCTCCTGGAATGC	GGTGACTGCTCTGTGTGCTGTAAC
mSTAT1	GGGGTACAGTTCACTGTCAAGTC	CCAGACTTCCGTTGGTGGATT
mSTAT2	GGACGTTCGACAGCATTTGG	CCCGGCTGAATTTTCGCAAG
mSTAT3	AGGCAAAGGGGAACACCTC	ACAGAGTGGGGCAACAGGTA
mSTAT4	GGAACACAAAGTGTCTGCCATTA	TCTTGCAGCAGTGTCAAAACTTCC
mSTAT5	CTTGAGGGGAACTCTTCGGG	GCGGTGGAGGCTGTTACTTC
mSTAT6	GGACTGCTTCCAGAGCACTT	AGACAGCGTTTGGTGAGGTC
mirf2-ChIP1	ACAAGGGCATGCATACTTTCCT	TACACCTGCTTCTTGCCTGG
mirf2-ChIP2	GCCGTCTTTTCAGCTGACTTC	GCTTAAGTGCTCAGAACCGC
mirf2-ChIP3	ACTGACGGGCTTTCATTTCCA	TGCCCTCGAGAGAAAACACA
mirf2-ChIP4	ACCTTGTCACTCGTTTGCGA	GTGGCAGAAAGCTAACCCCA
mPD-1-ChIP1	GAGGTCCTTTCACTCTCCACGG	CCCTGCCTTGCTCTGATGTTC
mPD-1-ChIP2	ATGGCAGACAAGGTAGGGGAGGGTC	CCAGGGCTGAGAGAGACTGAAAC
mPD-1-ChIP3	ACTGCTACTGAAGGCGACAC	ACCGAGGGTGAACGTTTCTT
mPD-1-ChIP4	GGGCATGTCCGCAAATGAAG	GGTGTCCCACGTTGAGCTAT
